# Breast cancer is a promising target for vaccination using cancer-testis antigens known to elicit immune responses

**DOI:** 10.1186/bcr1749

**Published:** 2007-07-24

**Authors:** Mark Taylor, Louise M Bolton, Peter Johnson, Tim Elliott, Nick Murray

**Affiliations:** 1 Cancer Research – UK Clinical Centre, University of Southampton, MP824, Southampton General Hospital, Tremona Road, Southampton SO16 6YD, UK

## Abstract

**Introduction:**

Cancer-testis antigens (CTAGs) are expressed solely in germ cells and in malignant tissues. They are targets of immune responses mediated by cytotoxic T cells in some cancers, and there is much interest in developing vaccines that induce these responses. The purpose of the present study was to ascertain the frequency of expression of CTAGs in breast cancer.

**Methods:**

Breast tumours were collected sequentially in the Southampton Tumour Bank from donors who had given written informed consent. Stored samples where there was sufficient material were sampled in sequence. An initial series of 42 tumours was screened for expression of 17 different CTAGs. A second panel of 40 tumours was screened for the expression of those antigens present in the first panel.

**Results:**

Ninety-three per cent of tumours in the first series expressed at least one CTAG, and 62% expressed the single antigen CTAG1. Eighty per cent of tumours in the second series expressed at least one CTAG, 50% expressing CTAG1. Tumours exhibiting higher risk features tended to express more CTAGs.

**Conclusion:**

More than two-thirds of breast cancers would be covered by a vaccine directed against just three CTAGs – CTAG1, BAGE1, and MAGEA10 – all of which are known to be targets of cytotoxic-T-lymphocyte responses.

## Introduction

The immune system has been demonstrated capable of mounting a number of different types of response against malignancy, either spontaneously or following immunisation. In some cases, the development of such responses has been associated with tumour regression.

Of particular interest are tumour antigens recognised through the class 1 antigen presentation system. Class-1-restricted (usually CD8-positive) cytotoxic T lymphocytes (CTL) recognise MHC class 1 molecules to which are bound short peptides derived from the intracellular breakdown of endogenous proteins. The antigen-specific T-cell receptor recognises the cell surface complex of peptide contained within the antigen-binding cleft of the class 1 molecule. Antigen recognition results in CTL killing of the antigen-bearing cell.

Understanding the process of assembly of the class 1 molecule-peptide antigen complex is crucial when considering strategies for tumour antigen discovery, and for assessing the presence of known tumour antigens in human cancers. The primary source of antigens for the MHC class I processing pathway is defective ribosomal products (that is, polypeptides that are only partially translated and are not folded; see, for example, Yewdell and colleagues [1]). These defective ribosomal products are rapidly degraded in the proteasome and transferred to the endoplasmic reticulum for assembly with class 1 molecules. Because defective ribosomal products are the antigen source, peptide antigens may be derived not just from those fully assembled proteins that are demonstrably present in the cell, but also from short fragments arising from translation of normally untranslated regions, introns or alternate reading frames; there are examples of effective recognition by CTL of cells in each of these cases. Assessment of what antigens might be available for presentation by the class 1 system in a cell is therefore best performed by analysis of RNA transcripts rather than by protein expression analyses.

A variety of human tumour antigens have now been identified. These antigens fall into several categories, of which the category cancer-testis antigens (CTAGs) has received particular attention [2]. These antigens are expressed in germ cells and in malignancies, but not at other sites. More than 15 families of genes encoding CTAGs have now been identified, and characterisation of these families has included demonstration that they are not expressed in normal somatic tissues.

Estimates of the frequency of expression of a particular CTAG in a particular tumour type have usually been made when such antigens were first described. Fewer systematic attempts to identify what proportion of any particular tumour type will express any CTAGs have been published. It is necessary, however, to establish whether or not such antigens are commonly found in a given tumour type to determine whether it is worth pursuing attempts at a vaccination strategy in that tumour.

Patterns of expression of some CTAGs have been reported in breast cancer by several authors [3-6]. In the current study we examined a larger panel of CTAGs in a series of 42 breast cancers, and then sought to confirm the pattern of distribution in a second series of 40 breast cancers collected at a later date. We report that several known CTL antigens are expressed frequently in breast cancer.

## Materials and methods

### Breast cancer cDNA

Frozen specimens of breast tumours were obtained from the Southampton Tumour Bank. All specimens had been obtained from individuals undergoing surgery for primary invasive breast cancer. Samples were identified and removed by a pathologist and were frozen as soon as possible. All specimens were taken from donors who had given prior written consent to their tumours being taken and stored in this way. Local research ethics committee approval has been given to the creation of the tumour bank, and local research ethics committee approval was obtained for this study.

Tumour samples were taken from the tumour bank in the order in which they had been stored – with the qualification that if only one aliquot of tumour was available, that sample was not used for this study.

Frozen tumour specimens were made into 5 μm sections using a cryostat (Bright Instrument Company, Huntingdon, UK) and the sections transferred to RNALater (Ambion (Europe) Ltd, Huntingdon, UK). These sections were then stored at -20°C until use. poly-A RNA was subsequently purified using oligo-dT magnetic beads (Dynal Biotech UK, Wirral, UK) according to the manufacturers' protocol. The RNA amount was standardised using ribogreen quantitation (Invitrogen Ltd, Paisley, UK).

First-strand cDNA was synthesised using M-MLV-H-Point mutant RT (Promega UK Ltd, Southampton, UK). The DNA quality was confirmed by an actin PCR. In every case, an equal amount of RNA was used in an equivalent reaction tube with all the other reagents added except for RT (RT-negative sample). RT-positive and RT-negative specimens were routinely used in all reactions to provide an adequate negative control.

### Primers

Primer sequences were selected using published sequence data and the Primer3 software [7].

For the MAGEA family, where possible, primers were designed to give coverage of more than one family member. Coverage of MAGEA1, MAGEA2, MAGEA3, MAGEA4, MAGEA5, MAGEA10, MAGEA11 and MAGEA12 was achieved. The results for these primers are presented in Tables 1 and 2 as positivity or negativity for the relevant group of antigens (data not shown for the uniformly negative findings with MAGEA1, MAGEA4 and MAGEA11).

**Table 1:** Expression of cancer-testis antigens by breast tumours in the first series

Sample	CTp11	SCP1	BAGE1	CTAG1	CTAG2	MAGEA10	MAGEA2312new^a^	MAGEA5	Number of cancer-testis antigens expressed
Number of positive samples	6	15	6	25	0	1	3	5	
1	-	-	-	-	-	-	-	-	0
2	-	-	-	-	-	-	-	-	0
3	+	-	-	+	-	-	+	+	4
4	-	+	+	-	-	-	-	-	2
5	-	-	-	+	-	-	-	-	1
6	-	-	-	-	-	-	-	-	0
7	-	-	-	+	-	-	-	-	1
8	-	+	-	-	-	-	-	-	1
9	-	-	+	+	-	+	+	+	5
10	-	-	-	+	-	-	-	-	1
11	-	-	-	+	-	-	-	-	1
12	-	+	-	+	-	-	-	-	2
13	-	-	-	+	-	-	-	-	1
14	+	-	-	+	-	-	-	-	2
15	-	+	-	-	-	-	-	-	1
16	-	-	-	+	-	-	-	-	1
17	-	+	-	-	-	-	+	+	3
18	-	-	-	+	-	-	-	-	1
19	-	+	+	+	-	-	-	-	3
20	+	+	+	-	-	-	-	-	3
21	-	+	-	-	-	-	-	-	1
22	-	+	-	-	-	-	-	-	1
23	+	-	+	+	-	-	-	-	3
24	-	-	-	+	-	-	-	-	1
25	-	-	-	-	-	-	-	-	0
26	-	-	-	+	-	-	-	-	1
27	-	+	-	+	-	-	-	-	2
28	-	+	-	-	-	-	-	-	1
29	-	-	-	-	-	-	-	-	0
30	-	-	-	-	-	-	-	-	0
31	-	-	-	+	-	-	-	-	1
32	+	+	-	+	-	-	-	-	3
33	-	+	-	-	-	-	-	-	1
34	-	+	-	+	-	-	-	-	2
35	-	-	-	+	-	-	-	+	2
36	+	-	-	+	-	-	-	-	2
37	-	+	+	-	-	-	-	-	2
38	-	-	-	+	-	-	-	-	1
39	-	-	-	+	-	-	-	-	1
40	-	-	-	+	-	-	-	-	1
41	-	-	-	-	-	-	-	+	1
42	-	-	-	+	-	-	-	-	1

-, negative result; +, positive result. ^a^The Primer pair MAGEA2312new recognises cDNA encoding MAGEA2, MAGEA3, and MAGEA12.

**Table 2:** Expression of cancer-testis antigens by breast tumours in the second series

Sample	CTp11	SCP1	BAGE1	CTAG1	CTAG2	MageA10	MageA2312new^a^	MAGEA5	Number of cancer-testis antigens expressed
Number of positive samples	18	8	4	20	5	6	1	2	
43	-	+	-	+	-	-	-	-	2
44	+	+	-	+	+	-	-	+	5
45	-	-	-	-	-	+	-	-	1
46	-	+	+	+	-	-	-	-	3
47	-	-	-	-	-	+	-	-	1
48	-	-	-	+	-	-	-	-	1
49	-	-	-	-	-	-	-	-	0
50	-	-	-	+	-	-	-	-	1
51	-	-	-	-	-	-	-	-	0
52	-	-	-	-	-	+	-	-	1
53	-	-	-	-	-	-	-	-	0
54	+	-	-	-	+	-	-	-	2
55	+	-	-	+	-	-	-	-	2
56	-	-	-	+	-	-	-	-	1
57	-	-	-	+	-	-	-	-	1
58	+	-	-	-	-	-	-	-	1
59	-	-	-	-	-	-	-	-	0
60	-	-	-	-	-	-	-	-	0
61	-	-	-	-	-	-	-	-	0
62	+	-	-	+	-	-	-	-	2
63	-	+	-	+	-	-	-	-	2
64	+	-	-	-	-	-	-	-	1
65	-	-	-	-	-	-	-	-	0
66	-	-	-	+	-	-	-	-	1
67	+	-	-	-	-	+	-	-	2
68	+	-	+	-	-	-	-	-	2
69	-	-	-	+	-	-	-	-	1
70	+	-	-	-	-	+	-	-	2
71	+	+	-	+	-	-	-	-	3
72	+	-	-	+	-	-	-	-	2
73	+	-	-	+	-	-	-	-	2
74	+	-	-	+	-	-	-	-	2
75	+	+	-	-	-	-	-	-	2
76	-	-	-	+	+	-	+	-	3
77	+	-	+	-	-	-	-	-	2
78	-	-	-	-	-	-	-	-	0
79	+	+	+	-	-	-	-	-	3
80	-	+	-	+	-	-	-	-	2
81	+	-	-	+	+	-	-	-	3
82	+	-	-	+	+	+		+	5

-, negative result; +, positive result. ^a^The Primer pair MAGEA2312new recognises cDNA encoding MAGEA2, MAGEA3, and MAGEA12.

Primers were designed to be intron spanning. Some of the primer pairs give a positive band with genomic DNA. In all such cases the band from genomic DNA was of a different size to that obtained from cDNA, and a genomic control was routinely included in all PCR experiments.

The specificity of each primer set was confirmed and the reaction conditions were defined by PCR with 50 pg plasmid DNA containing an IMAGE clone known to contain the relevant sequence [8]. Cycle numbers (between 30 and 40) were set to permit easy identification of a band on an agarose gel.

### IMAGE clones

Bacterial stocks transformed with plasmids containing the relevant image clones were obtained from MRC Geneservices (now known as Geneservice Ltd, Cambridge, UK). Bacteria were cultured and the plasmid DNA extracted.

### Polymerase chain reaction

The PCR was performed preferentially with Titanium Taq DNA polymerase (BD Biosciences, Oxford, UK). Accuprime DNA Polymerase (Invitrogen Ltd, Paisley, UK) was used when it was not possible to design primers fulfilling the stringent requirements of Titanium Taq. Preliminary experiments with a range of commercially available Taq DNA polymerases had shown that these two polymerases had the highest sensitivity and specificity when used with cDNA derived from frozen breast tumour specimens.

Depending on the primer pair combination, between 30 and 40 cycles of PCR were performed on RT-positive and RT-negative cDNA samples, together with a positive control of a plasmid containing the relevant cDNA. Water and genomic DNA were routinely included as negative controls. The use of a genomic DNA control removed the risk of false positives due to the existence of as yet unknown pseudogenes.

The results were scored as follows. Where the positive control yielded a product of the appropriate size, and all samples (both RT-positive and RT-negative) gave a negative result, the reaction was scored as negative for all breast cancer specimens and was not repeated. Where positive results were obtained in the RT-positive specimens but not in the RT-negative specimens, and the negative controls were negative, the reaction was repeated once, or in some cases twice. Where two reactions were performed, only those samples that gave a positive result in both reactions and where the negative controls were consistently negative were counted as positive samples. Where three reactions were performed, those specimens for which two out of three reactions gave a positive result but all the negative controls were negative were counted as positive samples.

### Adjuvant! Online

Patient and tumour characteristics were entered into the Adjuvant! Online version 7 programme [9] to obtain estimates of risk of breast cancer recurrence and of breast-cancer-specific mortality over 10 years [10].

## Results

Of 16 CTAGs examined in the first panel of 42 breast cancer samples, seven antigens were found not to be expressed in any of the specimens: SSX2, SSX4, CTAG2, CAGE1, MAGEA1, MAGEA4 and MAGEA11. The results for the other antigens are presented in Table 1.

Thirty-nine out of 42 (93%) tumours expressed at least one of the antigens examined, and 24/42 (57%) expressed two or more antigens. The most commonly expressed antigen was CTAG1, found in 62% of tumours. Sixty-seven per cent of tumours expressed at least one of CTAG1 or BAGE1, and 83% expressed at least one of CTAG1, BAGE1 or SCP1 (the same percentage of tumours was found to express at least one of CTAG1, CTp11 or SCP1).

Having identified a group of antigens and antigen combinations of potential interest in breast cancer, we then examined whether the findings were reproducible.

A second panel of 40 breast tumours was examined for the antigens expressed in the first series, and the pattern of expression was found to be very similar (Table 2), although not identical. Eighty per cent of tumours expressed at least one of the antigens, and 53% expressed two or more antigens. CTAG1 was again the most commonly expressed antigen (50%). Sixty per cent of specimens expressed at least one of CTAG1 or BAGE1, rising to 70% if MAGEA10 was also considered. Inclusion of SCP1 added nothing to the CTAG1/BAGE combination. Seventy-three per cent of samples expressed at least one of CTAG1 or CTp11 (SCP1 did not increase this proportion, although inclusion of MAGEA10 raised the expression to 80%).

Five of the specimens examined were from tumours found to contain only ductal carcinoma *in situ*. Three of these samples expressed one antigen (one each of CTAG1, CTp11 and SCP1), one sample expressed both CTp11 and BAGE1, and one sample expressed no CTAGs (although on review this specimen contained very little tumour).

**Figure 1:**
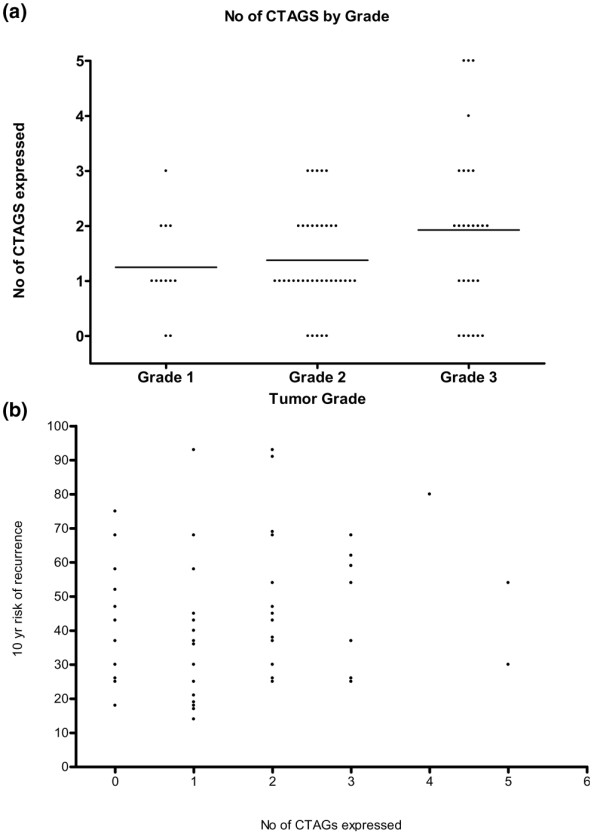
**(a) **Number of CTAGs expressed by tumor grade. **(b) **Calculated risk of recurrence by number of CTAGs expressed. CTAGs, cancer-testis antigens.

Known risk factors (oestrogen receptor status, tumour grade, nodal status and clinical stage) were examined to determine whether there was any correlation with the pattern of expression of CTAGs in the invasive cancers studied. There was a trend towards expression of more CTAGs with more aggressive features (for example, Figure 1a), but this did not achieve statistical significance.

The 10-year risk of recurrence or death from breast cancer was calculated for each patient using the Adjuvant! Online program. There was an apparent trend towards increased expression of CTAGs with increased risk, but this was not statistically significant (Figure 1b).

## Discussion

There is much interest in the potential for the use of vaccination against CTAGs in the treatment of solid human tumours. A limited number of reports have been published previously looking at breast cancer, but we have examined a larger panel of antigens than previously reported.

CTAG1 has been reported to be expressed in 10–42% of breast cancers in previous studies, with the highest frequency found in the largest study – 88 Japanese breast cancer tumours reported by Sugita and colleagues [11]. What the present report adds is that, in two separate series of tumours in the Southampton Tumour Bank, collected at different times, more than 80% of tumours were found to express at least one antigen.

Given that no individual antigen is expressed in all breast cancers, we were interested in examining the frequency of various combinations. In the first series of 42 tumours, 83% were found to express at least one of CTAG1, CTp11 or SCP1. Seventy-three per cent of tumours in the second series expressed at least one of these three antigens. There would be undoubted attraction in a polyvalent vaccine with coverage of a larger proportion of tumours than is possible with vaccines directed against a single antigen. Polyvalent vaccines containing multiple melanoma CTL antigens are already in clinical development.

T-cell responses, primarily CD8-mediated CTL responses, are believed to be the most important component of immune responses directed against CTAGs in human cancers. The initial descriptions of CTAGs were based on identification of antigens recognised by CTLs [12]. An alternative strategy, SEREX, has been used subsequently to identify potential CTAGs; thus far, no CTL responses have been reported against some CTAGs identified in this way. The antigens CTp11 and SCP1 both fall into this group of CTAGs for which no CTL responses have yet been reported. We therefore examined patterns of expression of CTAGs known to induce CTL in humans.

CTL responses have been identified against CTAG1, BAGE1 and MAGEA10 [13-15]. A vaccine directed against these three antigens would cover more than two-thirds of the tumours in each series of breast cancer examined in the present study. Construction of such a vaccine is entirely possible with currently available technology.

We did not analyse the HLA status of the individuals from whom the tumour specimens were derived, but have no reason to believe this would be different from that of the UK population at large. CTAG1 is the most studied of the three antigens with identified responses, and peptides have been identified as being presented by HLA A2, HLA A31, HLA B7, HLA B35, HLA B51, HLA Cw3 and HLA Cw6. The frequency of these HLA alleles in the general population ranges between 5% and 44% [16]. MAGEA10 peptides are known to be presented by HLA A2 (44%) and HLA B53 (2%). BAGE1 has been studied least, and there is only the initial report of its presentation by HLA Cw16 (7%). There is every reason to believe that other restriction elements (HLA alleles that present antigenic peptides) will be identified as further studies are undertaken. Analysis of the BAGE1 protein sequence using syfpeithi MHC epitope prediction software [17] identifies potential high-affinity peptide epitopes for HLA A2, HLA A3, HLA A11, HLA A24, HLA A26, HLA B8, HLA B18, HLA B44 and HLA B51. Similarly, an even larger number of potential high-affinity epitope and restriction element pairs exists for MAGEA10.

Clinical trials of immunisation against several CTAGs are underway. A search of the  website [18] identifies 14 current trials involving immunisation against CTAG1 or against MAGE. Although a number of trials of immunisation strategies in breast cancer are currently recruiting, we are not aware of any involving CTAGs. Our work, and that of others, would support inclusion of breast cancer patients in screening for entry into trials of vaccines against CTAGs.

An interesting question for the future will be, if vaccination can induce demonstrable CTL responses against CTAGs expressed in breast cancer, whom should be offered such vaccination? Early trials looking to establish clinical benefit are likely to be performed in individuals with advanced disease, perhaps when few other treatment options remain. Breast cancer, almost uniquely, is a condition where active management of metastatic disease can include long periods of treatment with nonimmunosuppressive therapies (hormonal agents, herceptin). There is therefore also an opportunity for quite prolonged vaccination schedules without the concern that the concomitant use of cytotoxic therapy will disrupt the evolution of a normal immune response. The setting of minimal residual disease following surgery and adjuvant therapies, however, may be where vaccination could have its greatest impact, perhaps increasing the number of long-term cures.

We examined whether there is an association between expression of CTAGs and markers of tumour aggressiveness. There was a trend towards higher numbers of CTAGs expressed with various features known to be associated with poorer prognosis. Similar findings were reported by Sugita and colleagues for CTAG1 [11], although in our dataset we could find no association for CTAG1 alone.

We entered the tumour features into the AdjuvantOnline programme, a web-based prognostication tool that offers predictions of likelihood of breast cancer recurrence and death over a 10-year period. There was a trend towards expression of greater numbers of CTAGs with both increased risk of recurrence and breast-cancer-specific mortality.

Park and colleagues [6] examined 12 breast cancers as part of a larger series also including lung and head and neck cancers. They found expression of at least one antigen of MAGEA1–MAGEA6 in 11/12 (91%) of samples. It is not clear whether this represents a population difference (all their samples were derived from Korean patients), represents an increased sensitivity of the nested PCR technique they used, or represents chance. The same group subsequently demonstrated that they could detect PCR evidence of circulating tumour cells in a proportion of patients with breast cancer [19]. Identification of circulating tumour cells may lead to earlier diagnosis of breast cancer, or to its recurrence. On the basis of the data presented here, the use of primers for CTAG1, MAGEA10 and BAGE1 might increase the sensitivity of such a test.

## Conclusion

The present article has shown that a large proportion of breast cancers express CTAGs. A vaccine directed against just three antigens known to be capable of eliciting CTL responses (CTAG1, MAGEA10 and BAGE1) would be of potential utility in up to 70% of breast cancers.

## Abbreviations

CTAG = cancer-testis antigen; CTL = cytotoxic T lymphocyte; HLA = human leucocyte antigen; MHC = major histocompatibility complex; PCR = polymerase chain reaction; RT = reverse transcriptase.

## Competing interests

The authors declare that they have no competing interests.

## Authors' contributions

MT and LMB contributed to experimental design and performed much of the experimental work. NM conceived the project and supervised the experimental work in conjunction with TE. PJ initiated the breast tumour specimen collection programme and participated in discussions on project design.
